# Draft genome sequence data of *Clostridium thermocellum* PAL5 possessing high cellulose-degradation ability

**DOI:** 10.1016/j.dib.2019.104274

**Published:** 2019-07-15

**Authors:** Eiko Nakazono-Nagaoka, Takashi Fujikawa, Ayumi Shikata, Chakrit Tachaapaikoon, Rattiya Waeonukul, Patthra Pason, Khanok Ratanakhanokchai, Akihiko Kosugi

**Affiliations:** a Biological Resources and Post-Harvest Division, Japan International Research Center for Agricultural Sciences (JIRCAS), Japan; b Institute of Fruit Tree and Tea Science, National Agriculture and Food Research Organization (NARO), Japan; c Pilot Plant Development and Training Institute (PDTI), King Mongkut's University of Technology Thonburi (KMUTT), Thailand; d Enzyme Technology Laboratory, School of Bioresources and Technology, King Mongkut's University of Technology Thonburi (KMUTT), Thailand

**Keywords:** *Clostridium thermocellum*, Cellulose, Cellulose-degradation, Draft genome sequence

## Abstract

*Clostridium thermocellum* is a potent cellulolytic bacterium. *C. thermocellum* strain PAL5, was derived from strain S14 that was isolated from bagasse paper sludge, possesses higher cellulose-degradation ability than representative strains ATCC27405 and DSM1313. In this work, we determined the draft genome sequence of *C. thermocellum* PAL5. Genomic DNA was used for whole-genome sequencing using the Illumina HiSeq 2500. We obtained 215 contigs of >200 bp (N50, 78,366 bp; mean length, 17,378 bp). The assembled data were subjected to the National Center for Biotechnology Information (NCBI) Prokaryotic Genome Annotation Pipeline, and 3198 protein-coding sequences, 53 tRNA genes, and 4 rRNA genes were identified. The data are accessible at NCBI (the accession number SBHL00000000). Our data resource will facilitate further studies of efficient cellulose-degradation using *C. thermocellum*.

**Table:** Specifications table

Subject area	*Biology*
More specific subject area	*Bacteriology, Genomics*
Type of data	*Genomic sequence, predicted genes and annotation of respective proteins, deposited in NCBI database and available by links provided within article*
How data were acquired	*Whole-genome sequencing using Illumina HiSeq 2500*
Data format	*Raw and analyzed*
Experimental factors	*Genomic DNA extracted from pure culture of Clostridium thermocellum* PAL5
Experimental features	*Genome sequencing, de novo assembly, gene prediction*
Data source location	*Tsukuba, Ibaraki, Japan*
Data accessibility	Deposited data are available at the National Center for Biotechnology Information (NCBI) under the accession number SBHL00000000 (https://www.ncbi.nlm.nih.gov/nuccore/SBHL00000000)
Related research article	*C. Tachaapaikoon, A. Kosugi, P. Pason, R. Waeonukul, K. Ratanakhanokchai, K.L. Kyu, T. Arai, Y. Murata, Y. Mori, Isolation and characterization of a new cellulosome-producing Clostridium thermocellum strain, Biodegradation 23 (1) (2012) 57–68.*

**Table:** 

**Value of the data** ∙ Clostridium thermocellum PAL5 having strong cellulose-degradation ability was derived from strain S14 that was isolated from bagasse paper sludge. ∙ Data on draft genome sequence of stain PAL5 can be used to search and characterize genes and enzymes regarding high cellulose-degradation ability. ∙ The comparison of genome sequence data between C. thermocellum strains gives an opportunity to understand a difference of cellulose degradation ability.

## 1 Data

**Fig. 1:**
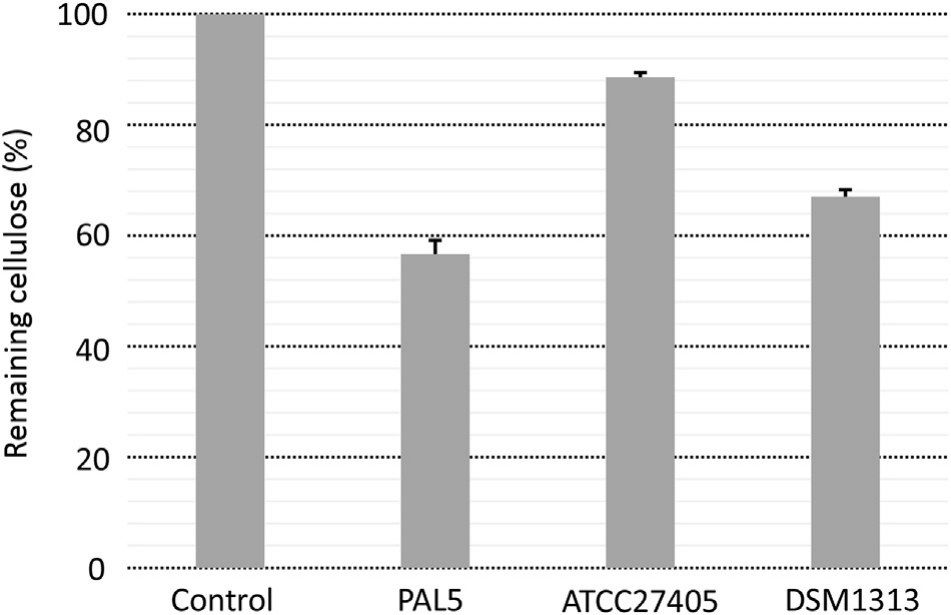
Comparison of cellulose-degradation ability of three strains of *Clostridium thermocellum*. The percentage of residual cellulose related to the original weight is shown for experiments with *Clostridium thermocellum* strains PAL5, ATCC27405, DSM1313 and uninoculated controls (control) after 3 days of incubation at 60 °C. PAL5, ATCC247405 and DSM 1313 were grown on CTFUD medium containing 1.0% microcrystalline cellulose. The data are means of four independent experiments. Error bars represent ± standard deviation (n = 4).

The thermophilic anaerobic bacterium *Clostridium thermocellum* (recently called *Hungateiclostridium thermocellum*) is a multifunctional ethanol producer, capable of both saccharification and fermentation [1]*. C. thermocellum* PAL5 was derived from strain S14 [2], [3], [4] that was isolated from bagasse paper sludge. The cellulolytic activity of strain PAL5 was compared with those of *C. thermocellum* ATCC27405^T^, a type strain of this species [5], and *C. thermocellum* DSM1313 [6] by incubation for 3 days at 60 °C in CTFUD medium [7] containing 1.0% microcrystalline cellulose powder instead of cellobiose. PAL5 showed better cellulose degrading ability than the other strains (Fig. 1), indicating that PAL5 may, like strain S14, possess high cellulose-degradation ability.

In this work, we determined the draft genome sequence of *C. thermocellum* PAL5 to identify which factors affect its cellulose-degradation ability. In total, 81,421,880 single reads with length 100 bp were obtained after filtering for quality score. Genome *de novo* assembly was performed using the CLC Genomic Workbench (CLC Bio, Qiagen, Valencia, CA); 215 contigs of >200 bp excluding scaffolded regions were obtained. Features of the genome are shown in Table 1. The assembled data for PAL5 were subjected to the NCBI Prokaryotic Genome Annotation Pipeline (PGAP), and 3,198 protein-coding sequences (CDSs), 53 tRNA genes, and 4 rRNA genes were identified. The equivalent values for strain ATCC27405 were 3,204 CDSs, 56 tRNA genes, and 12 rRNA genes (GenBank accession number:NC_009012). Thus, it was confirmed that the sequencing results for PAL5 in this work were similar to the known genome information for the type strain, and thus could be considered reliable.

**Table 1:** Features of *C. thermocellum* PAL5 genome.

Feature	Description
Number of reads used in assembly	81,421,088
Read length	100 bp
Genome size (total contig size)	3.84 Mbp
Assembly G + C percent	38.80%
N50 contig length	78,366 bp
Minimum contig length	208 bp
Maximum contig length	424,669 bp
Average contig length	17,378 bp
Number of contigs	215 contigs
Total contig size	3,736,353 bp
Genome coverage	2,178-fold

**Fig. 2:**
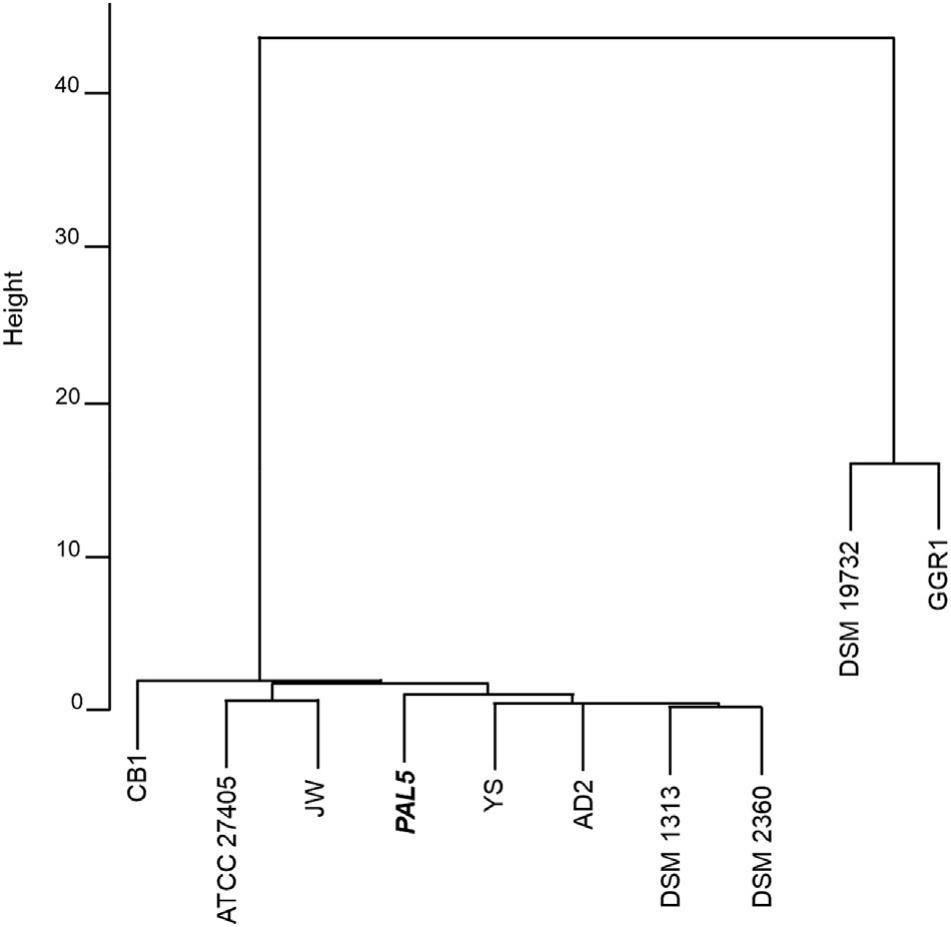
Dendrogram of average nucleotide identity (ANI) values. The ANI value for each combination of strains was calculated, and a dendrogram was constructed using the unweighted pair group method with arithmetic mean. *Clostridium clariflavum* DSM19732 (GenBank accession number: NZ_CP003065.1) and *Herbivorax saccinocola* GGR1 (NZ_CP025197.1) were used as outgroups. Strains of *Clostridium thermocellum*: PAL5, ATCC27405 (NC_009012), DSM1313 (NC_017,304), DSM2360 (NZ_CP016502), CB1 (NZ_CBQ0000000000.1), JW20 (NZ_ABVG00000000.2), AD2 (NZ_CP013828.1), and YS (AJGT00000000.1).

We used the average nucleotide identity (ANI) assay [8] among eight strains of *C. thermocellum*, including PAL5, and two out group strains, *C. clariflavum* DSM19732 (CP003065.1) and *Herbivorax* (*Hungateiclostridium*) *saccinocola* GGR1 (CP025197.1). The ANI value is calculated as the mean identity of BLASTn matches between the virtually fragmented query genome and the reference genome. A dendrogram of relatedness using ANI values (Suppl. Table 1) was constructed using the unweighted pair group method with arithmetic (UPGMA) method (Fig. 2) and single-linkage method (data not shown) as clustering methods, which showed that PAL5 is closely related to all the *C. thermocellum* strains*.*

Eight putative cellulosomal scaffolding protein of PAL5 were identified from genomic data by similarity with strain ATCC27405 (Table 2). The protein accession numbers corresponding to CipA and OlpB were divided into three nonconsecutive fragments; we suggest this was because the single reads could not be concatenated by the algorithm used in the *de novo* assembly. We consider that our genome data are of sufficient quality for further analysis to consider which factors affect the cellulose-degradation ability of strain PAL5 and others.

**Table 2:** Comparison of cellulosomal scaffolding proteins from strains ATCC27405^T^ and PAL5.

Predicted protein	ATCC27405^T^	Protein_accession number in PAL5
Scaffolding protein	CipA	THJ77199.1, THJ77201.1, THJ77215.1 (partial)
Anchoring protein	OlpA	THJ76703.1
	OlpC	THJ77790.1
	SdbA	THJ78951.1
	Orf2p	THJ76702.1
	OlpB	THJ76701.1, THJ77198.1, THJ77200.1 (partial)
Cellulosomal integrated protein	Cthe_0735	THJ78005.1
	Cthe_0736	THJ78004.1

## 2 Experimental design, materials, and methods

### 2.1 Genomic DNA extraction and sequencing

Genomic DNA of *C. thermocellum* PAL5 was extracted from microbial cells grown in anaerobic conditions at 60 °C. We used the cetyltrimethylammonium bromide (CTAB) method to extract genomic DNA [9]. The genomic DNA was processed to template samples using the TruSeq Nano DNA LT Library Prep Kit (Illumina, San Diego, CA). The template samples were formed into clusters using the HiSeq PE Rapid Cluster Kit v2-HS and HiSeq Rapid Due cBot v2 Sample Loading Kit, and then sequenced using the HiSeq Rapid SBS Kit v2-HS (Illumina) with the HiSeq 2500 next generation sequencer (Illumina). Genome *de novo* assembly was performed using the CLC Genomic Workbench. The assembled data were subjected to the NCBI PGAP.

### 2.2 Genomic average nucleotide identity

ANI analysis, which is used for *in silico* analysis of DNA–DNA hybridization, was performed. ANI values of combinations of the whole genome sequences of *C. thermocellum* strains were calculated using the web tool ANI calculator (http://enve-omics.ce.gatech.edu/ani/). The matrix made from ANI values between *C. thermocellum* strains was converted to a genetic dendrogram with algorithms such as the unweighted pair group method with arithmetic mean and single-linkage clustering method in the R statistic program.
